# Homing Pigeons and the Country Doctor

**Published:** 1900-03

**Authors:** 


### HOMING PIGEONS AND THE COUNTRY DOCTOR.

COUNTRY doctors do not lead the life of a sybarite, and anything lightening their burden is most grateful to them. The introduction of the home-made telephone, which, judging from the communications to a medical journal largely patronized by country practitioners, is in fairly general use, is of considerable assistance to them in the pursuit of their calling. But it is not always practicable for them to avail themselves of this method of communication with their patients. It is in such cases that the employment of a carrier-pigeon finds an eminent sphere of usefulness. A writer in the *Medical Counsel* describes his experience with these sure and fleet messengers and the great help they have been to him in his work. Carrier-pigeons are readily trained. A dove-cote may be constructed with electric connection with an annunciator from each window by means of which the bird's arrival is registered. The pigeons are marked by fastening a numbered ring around the leg. The doctor may leave two or more at the patient's house and can be informed of the progress of the case or be summoned when necessary, saving much speculation and uneasiness, and many unnecessary and wearisome trips. An editorial on this subject in *Pediatrics* quotes from the *Gazette des Hôpitaux* the success of a French writer with homing pigeons. It is surprising that this form of messenger service has not occurred before to the country doctor as being preferable to the hatless boy on horseback with his disconcerting tidings and maddening scarcity of details. The government maintains a cote of carrier-pigeons, and doubtless a line to the Department of the Interior would supply any one interested with all the desired information on this practical subject. W.

---

IF all the contributions to the literature of appendicitis during the last decade were collected in one volume, it would exceed in magnitude Webster's Dictionary or the illuminated family Bible. And still there is no agreement between the physician and surgeon. They are asymptotes, tending constantly to meet but never meeting. The physician points with the finger of pride to his array of grateful "recoveries" without operation, while the surgeon quotes the jingling aphorism "an inch and a half incision—a week and a half in bed."

As a compromise measure we have seen nothing more sensible and temperate than the following remarks delivered by Dr. Ochsner, of Chicago, before the Mississippi Valley Medical Association. Dr. Ochsner said:

> I have refrained from discussing the subject of appendicitis twice during this meeting. It is the first time I have ever done so when this subject was discussed in my presence, so that I hope the members will pardon me for saying a few words on it now.
>
> There is one point in regard to the treatment of this disease with which I am absolutely positive I have saved more lives than in all my practice as a surgeon. I may, therefore, be considered very enthusiastic in scattering this idea and impressing upon you a very important fact. The point is this: The appendix is located at a point at which, if it becomes inflamed and is not disturbed, it will be surrounded and cared for by the surrounding tissues. I have opened the abdomens of many hundreds of patients and have removed their appendices, so that what I may say is not against the surgical treatment, but is for the purpose of saving the lives of those that have appendicitis. The appendix becomes inflamed and immediately the omentum closes around the appendix; it shuts off the appendix. It does it every time. What happens? It is not disturbed, and we have catarrhal appendicitis; the catarrhal condition will subside. On the other hand, let us suppose the appendix is about to be perforated by a stone or foreign body; then the omentum will close in about it and we will have an abscess, but the abscess will be circumscribed. We will suppose, on the other hand, that we have an appendix in which the blood-vessels have been obliterated, giving us a gangrenous condition of the appendix; but it will be walled off from the general peritoneal cavity. What happens next? We give the patient milk or some other food, which immediately starts peristalsis; it draws the omentum away from the point and exposes the peritoneal cavity to general infection. Now, you cannot give this patient any kind of food but what the intestines will become distended with gas, and by increased peristaltic movements it will be forced through the ileo-cecal valve; it will disturb the conditions there. If we have an inflammation anywhere else, say an inflamed hand, we put it to rest. If we have an inflamed eye we generally close the eye. No matter of what part we have an inflammation we dispose of irritation. Why not do it in the case of the inflamed appendix? Why should we disturb the ileo-cecal valve and appendix by forcing gas through when it can be stopped? Of course, if a patient has eaten a large meal it will be forced through the ileo-cecal valve and out by the method described by Dr. Love. If the small intestine is to remain at rest you must not put a particle of food into the stomach and consequently the intestines will remain quiet; they will keep the omentum around there, and the worst that can happen will be a circumscribed abscess, and, if necessary, the general peritoneal cavity can be opened with safety. These patients can be given rectal alimentation, administering some of the concentrated foods, diluted with water, in the form of rectal enemas. After first washing out, the intestines should not be disturbed. I have made use of this method in over two hundred cases that were seen within the first few days of the attack, and so far there has been nothing but a circumscribed abscess. I have opened many cases and found a grangrenous appendix in a circumscribed abscess. I wish to impress upon you the fact that you cannot possibly harm a patient, whether you operate or not, by following this plan. If you will go home and follow it you will find many of your professional friends will do the same. Those who have tried this method inform me that their death-rate has been reduced greatly. I have used this method since 1892, but did not refer to it in public until about four years ago, and then not very enthusiastically, because I thought it was probably a foolish idea. Two years ago I began to bring this idea before the profession constantly, and since that time I have referred to it every time appendicitis was discussed. I would ask all of you to try it when you get a case of appendicitis, and it does not make any difference whether the patient has gastritis or enteritis. It is just as good and effective if he has a threatening ulcer of the stomach, or a threatening typhoid ulcer. You cannot do anything better for the patient; it is wonderful to see how quickly the pain will subside. Patients with gangrenous appendices have no pain after this treatment. I have kept patients for two or three weeks on rectal feeding and they get well. I have also taken patients with enormously distended abdomens, that were apparently hopeless cases, and after using this treatment they have recovered. I repeat that the worst that can follow this treatment is a circumscribed abscess.

---

THE Arlington Chemical Co., whose taste in art matters, as judged by the literature issued by them, is as elegant as their pharmaceutical products, are mailing to physicians a copy in colors of the beautiful painting, "The Country Doctor." The doctor has reached the house of his patient, and, overcoated and muffled, is trudging through the snow to the door where stands a woman with a lantern, its red glow sending a path of light across the snow. The doctor's jaded horse is seen with head hanging low, sending clouds of mist from his dilated nostrils. The color effects are fine and the motif of the picture can be appreciated by the country doctor.

---

NEWS comes of the promotion in the Philippine Islands of Dr. Luther B. Grandy. He has received the appointment of Major-Surgeon in the regular army. Dr. Grandy has been with the army in the Philippines for several months.

